# Benzaldehyde thio­semicarbazone monohydrate

**DOI:** 10.1107/S1600536808022769

**Published:** 2008-07-26

**Authors:** Sheng-Jiu Gu, Kai-Mei Zhu

**Affiliations:** a College of Pharmacy, Guilin Medical University, Guilin 541004, People’s Republic of China

## Abstract

In the title compound, C_8_H_9_N_3_S·H_2_O, intra­molecular N—H⋯N hydrogen bonding contributes to the mol­ecular conformation. Water mol­ecules are involved in inter­molecular N—H⋯O and O—H⋯S hydrogen bonds, which link the mol­ecules into ribbons extended along the *a* axis. Weak inter­molecular N—H⋯S hydrogen bonds link these ribbons into layers parallel to the *ab* plane with the phenyl rings pointing up and down.

## Related literature

For related crystal structures, see Beraldo *et al.* (2004 ▶); Bondock *et al.* (2007 ▶); Jing *et al.* (2006 ▶).
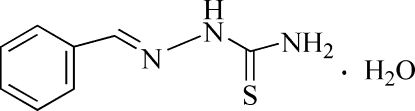


## Experimental

### 

#### Crystal data

C_8_H_9_N_3_S·H_2_O
*M* *_r_* = 197.26
Orthorhombic, 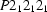

*a* = 6.1685 (10) Å
*b* = 7.6733 (12) Å
*c* = 21.131 (2) Å
*V* = 1000.2 (2) Å^3^
*Z* = 4
Mo *K*α radiation
μ = 0.29 mm^−1^
*T* = 298 (2) K
0.49 × 0.30 × 0.28 mm

#### Data collection

Bruker SMART CCD area-detector diffractometer
Absorption correction: multi-scan (*SADABS*; Sheldrick, 1996 ▶) *T*_min_ = 0.871, *T*_max_ = 0.923
4749 measured reflections
1764 independent reflections
1438 reflections with *I* > 2σ(*I*)
*R*_int_ = 0.065

#### Refinement

*R*[*F* ^2^ > 2σ(*F* ^2^)] = 0.045
*wR*(*F* ^2^) = 0.105
*S* = 1.07
1764 reflections
118 parameters
H-atom parameters constrained
Δρ_max_ = 0.24 e Å^−3^
Δρ_min_ = −0.17 e Å^−3^
Absolute structure: Flack (1983 ▶), 689 Friedel pairs
Flack parameter: −0.05 (13)

### 

Data collection: *SMART* (Siemens, 1996 ▶); cell refinement: *SAINT* (Siemens, 1996 ▶); data reduction: *SAINT*; program(s) used to solve structure: *SHELXS97* (Sheldrick, 2008 ▶); program(s) used to refine structure: *SHELXL97* (Sheldrick, 2008 ▶); molecular graphics: *SHELXTL* (Sheldrick, 2008 ▶); software used to prepare material for publication: *SHELXTL*.

## Supplementary Material

Crystal structure: contains datablocks I, global. DOI: 10.1107/S1600536808022769/cv2428sup1.cif

Structure factors: contains datablocks I. DOI: 10.1107/S1600536808022769/cv2428Isup2.hkl

Additional supplementary materials:  crystallographic information; 3D view; checkCIF report

## Figures and Tables

**Table 1:** Hydrogen-bond geometry (Å, °)

*D*—H⋯*A*	*D*—H	H⋯*A*	*D*⋯*A*	*D*—H⋯*A*
N3—H3*A*⋯N1	0.86	2.26	2.613 (4)	105
N2—H2⋯O1^i^	0.86	1.95	2.805 (3)	171
N3—H3*B*⋯S1^ii^	0.86	2.57	3.423 (3)	170
O1—H1*A*⋯S1	0.85	2.45	3.276 (2)	164
O1—H1*B*⋯S1^i^	0.85	2.44	3.284 (2)	172

Symmetry codes: (i) 
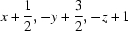
; (ii) 
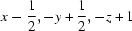
.

**Fig. 1.**
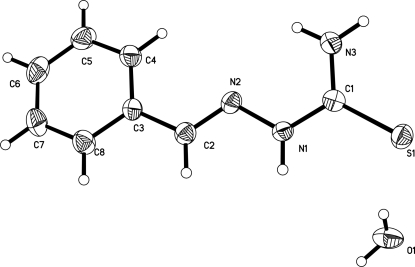
The content of asymmetric unit of the title compound showing the atomic numbering scheme and 30% probability displacement ellipsoids.

## supplementary crystallographic information

### Comment

Aryl-hydrazones, such as semicarbazones, thiosemicarbazones and guanyl hydrazones, exhibit strong biological activity. Therefor,they are important for drug design (Beraldo *et al.*, 2004),  organocatalysis and for the preparation of heterocyclic rings (Bondock *et al.*, 2007). In this paper, we present the title compound, (I).

In (I) (Fig. 1), the bond lengths and angles are normal and comparable to those observed in the reported compounds (Jing *et al.*, 2006). Intramolecular N—H···O hydrogen bond (Table 1) contributes to the molecular conformation. Crystalline water molecules are involved in the intermolecular N—H···O and O—H···S hydrogen bonds (Table 1), which link the molecules into ribbons extended along *a* axis. Weak intermolecular N—H···S hydrogen bonds (Table 1) link further these ribbons into layers parallel to *ab* plane with the up and down protruding phenyl rings.

### Experimental

Benzaldehyde (0.3 mmol) and thiosemicarbazide (0.3 mmol) were mixed in 50 ml flash in the presence of aqueous medium. After stirring 30 min at 373 K, the mixture then cooling slowly to room temperature and affording the title compound, then recrystallized from ethanol, affording the title compound as a colorless crystalline solid. Elemental analysis: calculated for C_8_H_11_N_3_OS: C 48.71, H 5.62, N 21.30%; found: C 48.58, H 5.65, N 21.24%.

### Refinement

All H atoms were placed in geometrically idealized positions (N—H 0.86, O—H 0.85 and C—H 0.93 Å) and treated as riding on their parent atoms, with *U*_iso_(H) = 1.2 *U*_eq_(C) (C,*O*,*N*).

### Crystal data

**Table:**

C_8_H_9_N_3_S·H_2_O	*D*_x_ = 1.310 Mg m^−^^3^
*M**_r_* = 197.26	Mo *K*α radiation λ = 0.71073 Å
Orthorhombic, *P*2_1_2_1_2_1_	Cell parameters from 1572 reflections
*a* = 6.1685 (10) Å	θ = 2.8–22.5º
*b* = 7.6733 (12) Å	µ = 0.29 mm^−^^1^
*c* = 21.131 (2) Å	*T* = 298 (2) K
*V* = 1000.2 (2) Å^3^	Block, orange
*Z* = 4	0.49 × 0.30 × 0.28 mm
*F*_000_ = 416	

### Data collection

**Table:**

Bruker SMART CCD area-detector diffractometer	1764 independent reflections
Radiation source: fine-focus sealed tube	1438 reflections with *I* > 2σ(*I*)
Monochromator: graphite	*R*_int_ = 0.065
*T* = 298(2) K	θ_max_ = 25.0º
φ and ω scans	θ_min_ = 1.9º
Absorption correction: multi-scan(SADABS; Sheldrick, 1996)	*h* = −7→7
*T*_min_ = 0.871, *T*_max_ = 0.924	*k* = −9→6
4749 measured reflections	*l* = −25→24

### Refinement

**Table:**

Refinement on *F*^2^	Hydrogen site location: inferred from neighbouring sites
Least-squares matrix: full	H-atom parameters constrained
*R*[*F*^2^ > 2σ(*F*^2^)] = 0.045	*w* = 1/[σ^2^(*F*_o_^2^) + (0.0438*P*)^2^ + 0.0825*P*] where *P* = (*F*_o_^2^ + 2*F*_c_^2^)/3
*wR*(*F*^2^) = 0.105	(Δ/σ)_max_ < 0.001
*S* = 1.08	Δρ_max_ = 0.25 e Å^−^^3^
1764 reflections	Δρ_min_ = −0.17 e Å^−^^3^
118 parameters	Extinction correction: none
Primary atom site location: structure-invariant direct methods	Absolute structure: Flack (1983), 689 Friedel pairs
Secondary atom site location: difference Fourier map	Flack parameter: −0.05 (13)

### Special details

**Table:**

Geometry. All e.s.d.'s (except the e.s.d. in the dihedral angle between two l.s. planes) are estimated using the full covariance matrix. The cell e.s.d.'s are taken into account individually in the estimation of e.s.d.'s in distances, angles and torsion angles; correlations between e.s.d.'s in cell parameters are only used when they are defined by crystal symmetry. An approximate (isotropic) treatment of cell e.s.d.'s is used for estimating e.s.d.'s involving l.s. planes.
Refinement. Refinement of *F*^2^ against ALL reflections. The weighted *R*-factor *wR* and goodness of fit *S* are based on *F*^2^, conventional *R*-factors *R* are based on *F*, with *F* set to zero for negative *F*^2^. The threshold expression of *F*^2^ > σ(*F*^2^) is used only for calculating *R*-factors(gt) *etc*. and is not relevant to the choice of reflections for refinement. *R*-factors based on *F*^2^ are statistically about twice as large as those based on *F*, and *R*- factors based on ALL data will be even larger.

### Fractional atomic coordinates and isotropic or equivalent isotropic displacement parameters (Å^2^)

**Table:**

	*x*	*y*	*z*	*U*_iso_*/*U*_eq_	
N1	0.5077 (4)	0.4006 (3)	0.35842 (11)	0.0430 (6)	
N2	0.4610 (4)	0.4442 (3)	0.42017 (10)	0.0402 (6)	
H2	0.5473	0.5107	0.4412	0.048*	
N3	0.1572 (5)	0.2820 (4)	0.41293 (12)	0.0616 (9)	
H3A	0.1923	0.2576	0.3746	0.074*	
H3B	0.0400	0.2401	0.4289	0.074*	
O1	0.2807 (3)	0.8582 (3)	0.51847 (12)	0.0681 (7)	
H1A	0.2874	0.7483	0.5138	0.082*	
H1B	0.4026	0.9030	0.5091	0.082*	
S1	0.22368 (12)	0.43397 (10)	0.52322 (3)	0.0473 (3)	
C1	0.2821 (5)	0.3831 (3)	0.44708 (14)	0.0398 (7)	
C2	0.6891 (5)	0.4535 (4)	0.33743 (13)	0.0436 (7)	
H2A	0.7840	0.5112	0.3645	0.052*	
C3	0.7511 (4)	0.4251 (4)	0.27170 (12)	0.0412 (7)	
C4	0.6111 (6)	0.3517 (4)	0.22810 (15)	0.0534 (9)	
H4	0.4745	0.3146	0.2409	0.064*	
C5	0.6728 (7)	0.3333 (5)	0.16566 (16)	0.0640 (11)	
H5	0.5766	0.2870	0.1362	0.077*	
C6	0.8751 (7)	0.3834 (5)	0.14733 (17)	0.0636 (11)	
H6	0.9180	0.3671	0.1056	0.076*	
C7	1.0158 (6)	0.4572 (5)	0.18932 (16)	0.0630 (10)	
H7	1.1523	0.4940	0.1762	0.076*	
C8	0.9527 (5)	0.4761 (4)	0.25132 (15)	0.0536 (9)	
H8	1.0490	0.5248	0.2802	0.064*	

### Atomic displacement parameters (Å^2^)

**Table:**

	*U*^11^	*U*^22^	*U*^33^	*U*^12^	*U*^13^	*U*^23^
N1	0.0480 (15)	0.0477 (15)	0.0332 (13)	0.0000 (13)	0.0012 (12)	−0.0020 (11)
N2	0.0443 (14)	0.0422 (14)	0.0342 (12)	−0.0074 (14)	−0.0004 (11)	−0.0034 (12)
N3	0.061 (2)	0.079 (2)	0.0456 (17)	−0.0290 (17)	0.0118 (14)	−0.0121 (15)
O1	0.0524 (15)	0.0609 (13)	0.0910 (19)	0.0017 (12)	0.0204 (15)	−0.0130 (13)
S1	0.0467 (5)	0.0576 (5)	0.0376 (4)	0.0009 (4)	0.0015 (4)	−0.0018 (4)
C1	0.0402 (17)	0.0381 (16)	0.0411 (16)	0.0008 (15)	−0.0035 (15)	0.0036 (12)
C2	0.0415 (17)	0.0469 (17)	0.0422 (16)	0.0035 (17)	−0.0009 (14)	−0.0018 (14)
C3	0.0421 (17)	0.0433 (14)	0.0383 (15)	0.0013 (18)	0.0036 (14)	0.0015 (14)
C4	0.056 (2)	0.061 (2)	0.0429 (19)	−0.0134 (17)	0.0061 (17)	−0.0012 (17)
C5	0.083 (3)	0.065 (2)	0.044 (2)	−0.013 (2)	0.0033 (19)	−0.0067 (18)
C6	0.086 (3)	0.061 (2)	0.043 (2)	0.005 (2)	0.017 (2)	0.0038 (18)
C7	0.053 (2)	0.080 (3)	0.057 (2)	−0.002 (2)	0.0154 (18)	0.010 (2)
C8	0.050 (2)	0.066 (2)	0.0446 (17)	−0.0061 (17)	0.0023 (16)	0.0054 (16)

### Geometric parameters (Å, °)

**Table:**

N1—C2	1.270 (3)	C3—C8	1.373 (4)
N1—N2	1.378 (3)	C3—C4	1.382 (4)
N2—C1	1.327 (3)	C4—C5	1.380 (4)
N2—H2	0.8600	C4—H4	0.9300
N3—C1	1.310 (4)	C5—C6	1.362 (5)
N3—H3A	0.8600	C5—H5	0.9300
N3—H3B	0.8600	C6—C7	1.364 (5)
O1—H1A	0.8499	C6—H6	0.9300
O1—H1B	0.8499	C7—C8	1.375 (5)
S1—C1	1.695 (3)	C7—H7	0.9300
C2—C3	1.457 (4)	C8—H8	0.9300
C2—H2A	0.9300		
			
C2—N1—N2	115.9 (3)	C4—C3—C2	122.2 (3)
C1—N2—N1	119.6 (2)	C5—C4—C3	120.4 (3)
C1—N2—H2	120.2	C5—C4—H4	119.8
N1—N2—H2	120.2	C3—C4—H4	119.8
C1—N3—H3A	120.0	C6—C5—C4	119.7 (4)
C1—N3—H3B	120.0	C6—C5—H5	120.2
H3A—N3—H3B	120.0	C4—C5—H5	120.2
H1A—O1—H1B	109.4	C5—C6—C7	121.0 (3)
N3—C1—N2	117.6 (3)	C5—C6—H6	119.5
N3—C1—S1	122.3 (2)	C7—C6—H6	119.5
N2—C1—S1	120.1 (2)	C6—C7—C8	118.9 (3)
N1—C2—C3	121.1 (3)	C6—C7—H7	120.5
N1—C2—H2A	119.5	C8—C7—H7	120.5
C3—C2—H2A	119.5	C3—C8—C7	121.7 (3)
C8—C3—C4	118.2 (3)	C3—C8—H8	119.2
C8—C3—C2	119.6 (3)	C7—C8—H8	119.2

### Hydrogen-bond geometry (Å, °)

**Table:**

*D*—H···*A*	*D*—H	H···*A*	*D*···*A*	*D*—H···*A*
N3—H3A···N1	0.86	2.26	2.613 (4)	105
N2—H2···O1^i^	0.86	1.95	2.805 (3)	171
N3—H3B···S1^ii^	0.86	2.57	3.423 (3)	170
O1—H1A···S1	0.85	2.45	3.276 (2)	164
O1—H1B···S1^i^	0.85	2.44	3.284 (2)	172

Symmetry codes: (i) *x*+1/2, −*y*+3/2, −*z*+1; (ii) *x*−1/2, −*y*+1/2, −*z*+1.
